# Hemorrhagic Meningitis Due to Varicella-Zoster Virus: A Case Report With Clinical, Cytologic, and Radiologic Correlation

**DOI:** 10.7759/cureus.31113

**Published:** 2022-11-05

**Authors:** Vineet Gorolay, David Martens, Laveniya Satgunaseelan, Louise Van Camp, Gemma Winkler

**Affiliations:** 1 Department of Radiology, Royal Prince Alfred Hospital, Sydney, AUS; 2 Department of Rheumatology, Royal Prince Alfred Hospital, Sydney, AUS; 3 Department of Neuropathology, Royal Prince Alfred Hospital, Sydney, AUS

**Keywords:** vzv, mri, hemorrhagic meningitis, subarachnoid hemorrhage, meningitis, zoster, varicella

## Abstract

Although varicella-zoster virus (VZV) is known to affect the central nervous system in a protean manner, hemorrhagic VZV meningitis has not been well documented in the literature. Here, we correlate the clinical, cytologic, and radiologic findings in an immunocompromised patient presenting with subarachnoid hemorrhage associated with VZV meningitis. Clinical findings included multidermatomal zoster, myelitis, and neurapraxia. Magnetic resonance imaging findings included superficial siderosis and diffused linear and nodular leptomeningeal enhancement. Cerebrospinal fluid cytology revealed hemorrhage and lymphocytic pleocytosis. This case report adds hemorrhagic meningitis to the spectrum of complications associated with disseminated VZV infection.

## Introduction

Varicella-zoster virus (VZV) is known to affect the central and peripheral nervous system in a protean manner [1,2]. In immunocompromised patients, meningitis and myelitis may have atypical presentations and require a high index of suspicion for timely diagnosis [2]. VZV vasculopathy commonly affects large vessels and is associated with ischemic or hemorrhagic infarct [3], aneurysm, and intracranial hemorrhage [4]. When described, subarachnoid hemorrhage occurs in the setting of hemorrhagic infarct [5] or multifocal intraparenchymal hemorrhage [6]. Conversely, hemorrhagic meningitis manifesting predominantly as subarachnoid hemorrhage is poorly described in the literature. Here, we correlate the clinical, cytologic, and imaging findings in an immunocompromised patient presenting with hemorrhagic meningitis, multidermatomal zoster, myelitis, and neurapraxia.

## Case presentation

**Figure 1:**
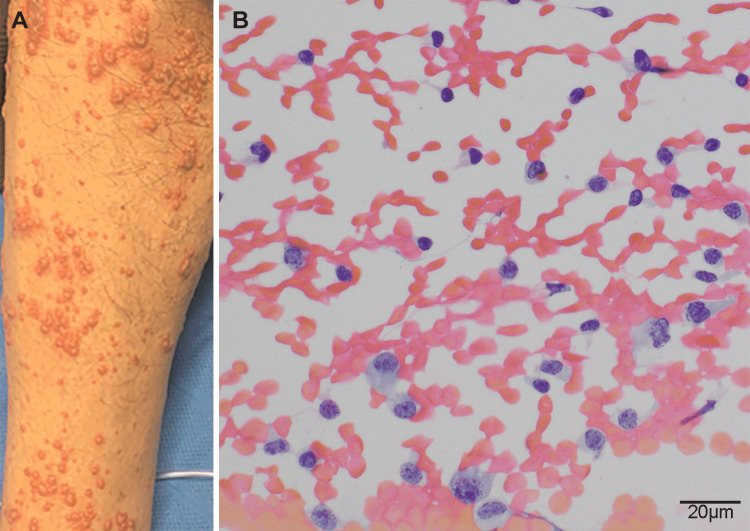
Zoster exanthem and cerebrospinal fluid cytology. A. Clinical photograph of the right leg demonstrating the typical vesicular rash of herpes zoster, with erythematous nodules and confluent lesions with crusting. B. Cerebrospinal fluid cytology Papanicolaou stain demonstrating lymphocytic pleocytosis typical of zoster meningitis. Small, mature lymphocytes and monocytes are admixed with enlarged lymphocytes with atypical nuclear features. Innumerable red cells correspond to the subarachnoid blood products seen on magnetic resonance imaging.

A 53-year-old man presented to our emergency department with 10 days of painful right leg rash and intermittent fevers. Pertinent background history included dermatomyositis complicated by polyarthritis and interstitial lung disease, treated with tacrolimus and mycophenolate and 25 mg prednisolone. He had recently ceased intravenous cyclophosphamide treatment. He was a non-smoker, with no history of hypertension, excess alcohol consumption, diabetes, or bleeding diatheses, and was not on antiplatelet or anticoagulant therapy. Human immunodeficiency virus status was negative. There was no documented history of VZV vaccination. Examination revealed a unilateral vesicular rash with confluent lesions and crusting along the L4 and L5 dermatomes of the medial right leg (Figure 1, Panel A). A positive VZV polymerase chain reaction (PCR) swab confirmed herpes zoster, and intravenous acyclovir was commenced. Prednisolone was continued, and tacrolimus and mycophenolate were withheld.

**Figure 2:**
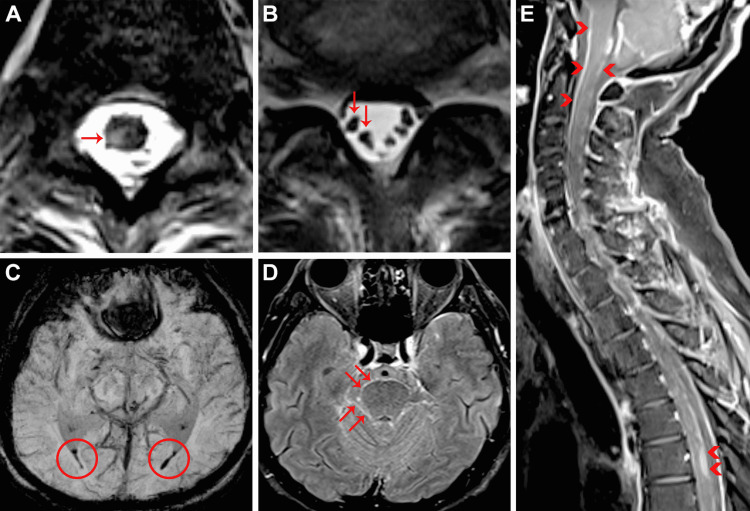
Neuroimaging features of zoster myelitis and hemorrhagic meningitis. A. Axial T2-weighted sequence through the thoracic spinal cord demonstrating a hyperintense lesion in the right dorsolateral cord (horizontal arrow). The same lesion enhanced following gadolinium administration (not shown). B. Axial T2-weighted sequence through the lumbar spine showing clumped and enlarged cauda equina nerve roots (vertical arrows). Confluent surface enhancement was present (not shown). C. Axial susceptibility-weighted minimum-intensity projection composite sequence through the supratentorial brain demonstrating blooming in the occipital horns of the lateral ventricles (circles). D. Axial post-gadolinium fluid-attenuated inversion recovery sequence at the level of the superior pontine tegmentum demonstrating florid leptomeningeal enhancement coating the pial surfaces of the pons and cerebellar folia (oblique arrows). E. Sagittal post-gadolinium T1 fat-suppressed sequence demonstrating marked enhancement along the ventral and dorsal aspect of the brainstem and spinal cord with plaque-like enhancement in the upper thoracic spinal cord (arrowheads).

Approximately one week later, the patient developed acute profound right lower limb weakness. He did not complain of photophobia or nuchal rigidity; Kernig and Brudzinski signs were negative. Magnetic resonance imaging (MRI) revealed focal hyperintensity and enhancement at the right dorsal horn of the lower thoracic cord (Figure 2A). Diffuse linear and nodular leptomeningeal thickening and enhancement were present along lower cranial nerves, cerebellar folia, brainstem, spinal cord, and cauda equina roots (Figures 2B, 2D, 2E). Susceptibility artifact associated with dependent fluid in the lateral ventricles (Figure 2C) and over the leptomeninges was also noted, consistent with subarachnoid blood products. No intraparenchymal hemorrhage or infarction was evident on MRI. No vessel wall irregularity, stenosis, aneurysm, or abnormal vessel wall enhancement was detected on computed tomography (CT) angiography or MRI.

Subsequent lumbar puncture revealed turbid red cerebrospinal fluid (CSF) in all three samples. Cytology revealed an increased number of leukocytes, including mature lymphocytes and monocytes, on a bloodstained background. A proportion of the lymphocytes demonstrated atypical morphology, characterized by increased amounts of cytoplasm, enlarged nuclei with irregular nuclear membranes, and coarse chromatin (Figure 1, Panel B).

The CSF protein was elevated, and CSF glucose was at the upper limits of normal, with an elevated white cell count and markedly elevated red cell count (Table 1). CSF PCR detected VZV DNA, without evidence of other infectious causes. An 18F-fluorodeoxyglucose positron emission tomography/computed tomography study did not detect evidence of occult malignancy.

**Table 1:** Laboratory parameters obtained from lumbar puncture CSF specimen. CSF: cerebrospinal fluid; PCR: polymerase chain reaction

CSF parameter (units)	Patient value	Reference range
Protein (g/L)	4.53	0.15–0.45
Glucose (mmol/L)	4.4	2.5–4.5
White cell count (×10^6^)	50	<5
Red cell count (×10^6^)	82,800	0
Varicella PCR	Detected	Not detected
Tuberculosis PCR	Not detected	Not detected
Culture	No growth	No growth
Cytology	No malignant cells detected	No malignant cells detected

The unifying diagnosis was of disseminated zoster with hemorrhagic meningitis, myelitis, and neurapraxia. Intravenous acyclovir was continued for a total duration of three weeks, and pregabalin was added for the treatment of neuropathic pain. The zoster lesions crusted over and resolved. The patient’s leg weakness improved after several months of rehabilitation to his baseline of symmetric weakness due to dermatomyositis. However, his post-herpetic neuralgia persisted at the one-year follow-up.

## Discussion

VZV is a prevalent infection responsible for varicella (chickenpox) and herpes zoster (shingles) [1] and a spectrum of central nervous system (CNS) complications, especially in immunocompromised patients [2]. Following primary infection, VZV establishes latency in the sensory dorsal root ganglion along the entire neuraxis by retrograde axonal transport or viremia [4]. During reactivation, antegrade axonal transport from infected sensory ganglia results in skin replication and a vesicular rash (zoster) involving one to three dermatomes [7].

The pathologic hallmarks of VZV include ganglionic hemorrhage, necrosis, and inflammation; demyelination can be seen with mononuclear infiltrates and microglial proliferation [7]. Detection of herpesvirus particles, Cowdry A inclusions, and multinucleated giant cells in involved tissues confirms viral invasion [4].

Meningitis and other CNS complications arise in a retrograde manner from infected dorsal root ganglia [2]. CSF cytology reveals lymphocytic pleocytosis [2], as evident in our case. Anti-VZV immunoglobulin G has been described as more robust than viral DNA PCR which disappears after early infection [2,6,8]. Post-gadolinium fluid-attenuated inversion recovery MRI sequences may demonstrate leptomeningeal enhancement with 80-90% sensitivity, attributed to pathogen-induced blood-brain-barrier dysfunction and leakage of blood-borne proteins into CSF [9]. Although imaging is not required to diagnose meningitis, it plays an important role in the assessment of complications including myelitis and vasculopathy.

VZV myelitis may occur via direct infection, vasculopathy, or as a post-infectious phenomenon, and may be fatal in immunocompromised patients [2]. MRI features of VZV myelitis include focal signal hyperintensity on T2-weighted sequences and intense gadolinium enhancement in the dorsolateral spinal cord; often at a corresponding level to the dermatomal rash [1].

Subarachnoid hemorrhage is a rarely described complication of VZV infection. A recent literature review identified ischemic stroke, intracerebral hemorrhage, and venous sinus thrombosis as the most common cerebrovascular complications following VZV infection [8]. Intraparenchymal hematoma with coexistent subarachnoid hemorrhage has been described and attributed to VZV vasculopathy [5,6]. VZV vasculopathy causes a large-vessel vasculitis [3], resulting in isolated or multifocal ischemic infarcts, [6] dissection, aneurysm, or stenoses of the terminal internal carotid artery or proximal circle of Willis [1]. However, CT angiography was normal in our case. No infarct, aneurysm, or vessel wall enhancement was detected on MRI. In the setting of superficial siderosis and nodular leptomeningeal enhancement, a hemorrhagic form of meningitis was considered most likely.

The pathogenesis of subarachnoid hemorrhage in our case could be due to a direct neurotropic effect as hemorrhagic necrosis is a hallmark of zoster [7]. Alternatively, small-vessel inflammation representing a form of small-vessel vasculopathy could be entertained [4]. Although ventriculitis is a described complication of VZV [3], it would not account for the extraventricular nodular leptomeningeal enhancement seen in our case.

Prompt treatment with steroids and intravenous acyclovir remains the first-line therapy for CNS complications of VZV [6]. The minimum suggested duration of therapy is two weeks, although immunocompromised patients may benefit from a longer treatment course [2]. Further prospective research is required to better inform treatment duration and dosing.

## Conclusions

Our case report adds hemorrhagic meningitis to the spectrum of complications of VZV reactivation, providing clinical, cytologic, and radiologic correlation. The disseminated pattern of disease in our case also included multidermatomal zoster, myelitis, neurapraxia, and post-herpetic neuralgia. In immunocompromised patients presenting with zoster, early empiric antiviral therapy and expedited investigation for disseminated infection are essential. Although gadolinium-enhanced MRI of the entire neuraxis is a useful tool to assess for disseminated disease, CSF analysis remains the gold standard for diagnosis of CNS involvement of VZV.
